# Vision comorbidities analysis for dementia risk prediction in 'electronic health records' across UK Biobank and All of US datasets

**DOI:** 10.1002/alz70860_100295

**Published:** 2025-12-23

**Authors:** Victoria Mery, Chloe Walsh, Antigone Fogel, Ramin Nilforooshan, Paresh Malhotra, Payam Barnaghi

**Affiliations:** ^1^ Imperial College London, London, United Kingdom; ^2^ UK Dementia Research Institute, Care Research and Technology Centre, London, United Kingdom; ^3^ UK Dementia Research Institute, Care Research and Technology Centre, Imperial College London, London, United Kingdom; ^4^ Surrey and Borders Partnership NHS Foundation Trust, Leatherhead, United Kingdom; ^5^ University of Surrey, Guildford, United Kingdom

## Abstract

**Background:**

Untreated vision loss has been identified as a late life modifiable risk factor of dementia. The association of vision impairment (VI) and cognitive deficit in ageing is yet to be fully understood.

**Method:**

In this case‐control analysis, the vision comorbidities of two dementia cohorts (UK Biobank *n* = 11721, All of US *n* = 2350) were analysed using electronic health records in ‘International Classification of Disease‐10’ (ICD‐10) format. Mann Whitney‐U analysis compared how dementia patients differed to controls with regards of pre‐defined blocks of VI conditions. VI diagnostic block counts and age of first diagnosis of VI were used to understand prevalence, pattern occurrences and severity of impairments.

**Result:**

‘Visual disturbances and blindness’ counts showed a small but notable increase in the dementia group compared to controls in UKB (*r* = 0.034, *p* <0.0001) and AoUS (*r* = 0.15, *p* <0.0001). Earlier diagnoses of ‘muscles, movement, accommodation and refraction’ (*r* = ‐0.16, *p* <0.001) and ‘disorders of the lens’ (*r* = ‐0.11, *p* <0.001) VIs were also more associated with dementia patients. Time between first VI diagnosis and dementia onset was shorter in Alzheimer's disease compared to Vascular dementia (*p* <0.001), but no other within‐group differences were seen.

**Conclusion:**

Our findings demonstrated a significant association between diagnosis of selected VI groups and dementia and support early management of VI conditions for its prevention.

